# Antibody-drug conjugate (disitamab vedotin) therapy targeting HER2-low or higher advanced extramammary Paget’s disease

**DOI:** 10.1093/oncolo/oyaf063

**Published:** 2025-05-27

**Authors:** Sheng Zhang, Juan Zhou, Xiaoying Zhao, Hualei Gan, Wenxia Peng, Wenfeng Li, Mingjuan Sun, Jiong Hu, Wangjun Yan

**Affiliations:** Medical Oncology, Shanghai Cancer Center, Fudan University, Shanghai, China; Medical Oncology, Shanghai Cancer Center, Fudan University, Shanghai, China; Medical Oncology, Shanghai Cancer Center, Fudan University, Shanghai, China; Pathology, Shanghai Cancer Center, Fudan University, Shanghai, China; Medical Oncology, Shanghai Cancer Center, Fudan University, Shanghai, China; Breast surgery, the affiliated hospital of Qingdao Universisty, Qingdao, China; Department of Biochemistry and Molecular Biology, College of Basic Medical Sciences, Naval Medical University, Shanghai, China; Medical oncology, RenJi Hospital, Shanghai Jiao Tong University, School of Medicine, Shanghai, China; Musculoskeletal Oncology, Shanghai Cancer Center, Fudan University, Shanghai, China

**Keywords:** extramammary Paget’s disease, antibody-drug conjugate, HER2

## Abstract

**Background and Objective:**

Metastatic Extramammary Paget’s disease (EMPD) is an extremely rare cancer and has a dismal prognosis. The rarity of metastatic EMPD has made the clinical trial almost unfeasible, thus high-quality retrospective analysis is valuable.

**Methods:**

The electronic medical files of patients with metastatic EMPD treated from Jan 2017 to April 2024 in a tertiary cancer center in China were reviewed, and available tissues were collected for HER2 staining, if possible. Eleven patients were treated with the anti-HER2 antibody-drug conjugate Disitamab vedotin (DV), using doses of 2 mg/kg, once every 3 to 4 weeks. The efficacy and toxicity data were extracted. Key Findings and Limitations: Treatment with the antibody-drug conjugate DV elicited an objective response in 8 of 11 patients (73%), and CEA response in 10 of the 11 patients (91%). The median progression-free interval was 5.5 months. The median overall survival was 21.9 months. This efficacy was accompanied by mild grade 1 or 2 treatment-related toxicity and no grade 3 toxicity. This study is limited by a small sample size, retrospective nature, and inevitable selection bias. Conclusions and Clinical

**Implications:**

Antibody-drug conjugate targeting of HER2 might be an active treatment for metastatic EMPD. We have initiated a clinical trial to confirm it prospectively.

**Implication for practice:**

Metastatic Extramammary Paget’s disease (EMPD) is an extremely rare cancer without standard treatment. Treatment with the antibody-drug conjugate DV (an anti-HER2 antibody-drug conjugate) elicited an objective response in 8 of the 11 patients (73%), and CEA response in 10 of the 11 patients (91%). This efficacy was accompanied by mild treatment-related toxicity. Antibody-drug conjugate targeting of HER2 might be an active treatment for metastatic EMPD.

## Introduction

Extramammary Paget’s disease (EMPD) is a rare neoplasm that usually develops in the apocrine gland-bearing areas of older adults.^1^ The prevalence of EMPD in China was reported to be 0.4 per million people in 2016,^2^ and the prevalence in Europe was 0.7 per million in 2012.^3^

Metastatic EMPD, especially in penoscrotal areacomprises a very small subset of EMPD that is incurable and has a dismal prognosis. Because of its rarity, few prospective trials evaluating treatments have been undertaken, and no standard of care has been established.^4^

It has been shown that the tumor cells express HER2 in 30% to 60% of patients with EMPD. In some case series, (mostly with 1 or 2 patients) anti-HER2 therapy with trastuzumab has shown antitumor activity, mostly in combination with chemotherapy.^3,5-11^

Antibody-drug conjugates are designed for selective delivery of potent cytotoxic drugs to antigen-expressing tumor cells by linking cytotoxins to monoclonal antibodies (mAbs). Disitamab vedotin (DV) is a humanized anti-HER2 antibody conjugated with monomethyl auristatin E (MMAE) via a cleavable linker. It has been approved for the treatment of gastric cancer and urothelial cancer and has been commercially available in China since July, 2021.^12^

Here we reviewed retrospectively patients with metastatic EMPD treated in our center, who were given an antibody-drug conjugate targeting HER2.

## Methods

All patients presented to Shanghai Cancer Center, Fudan University. They were diagnosed with histologically confirmed recurrent/metastatic EMPD. The electronic medical files of patients with metastatic EMPD from Jan 2017 to April 2024 were reviewed, and available tissues were collected for HER2 staining, if possible. Eleven patients were treated with the anti-HER2 antibody-drug conjugate DV, using doses of 2 mg/kg, once every 3 to 4 weeks. We provided here the efficacy data of DV treatment for this extremely rare cancer.

## Treatment procedure

The ethics committees of Fudan University Affiliated Shanghai Cancer Center approved this study.

Eight of the eleven patients have already received multiple lines of chemotherapies. (median 2). In addition to the relatively popular lymph node metastases, 9 of the 11 patients have visceral metastases.

The response of patients with measurable target lesions on CT imaging was evaluated according to RECIST version 1.1. The efficacy of those with skin lesions deemed as not measurable was evaluated according to WHO criteria.^13^ Clinical response was assessed by comparing pairs of clinical photographs obtained before and after treatment. The pairs of photographs were graded as follows:

• Complete response (CR) = reversion to clinically normal-appearing skin.

• Partial response (PR) = 50% or greater reduction in the longest diameter of the affected skin lesion.

• Progression of disease (PD) = 50% or greater increase in diameter of affected skin.

Progression-free survival (PFS) was defined as the time from initiation of DV treatment to first documentation of objective tumor progression or to death due to any cause, whichever occurred first. Overall survival was defined as the time from DV treatment initiation to death due to any cause.

### HER-2 test

HER2 IHC staining was performed with a Ventana BenchMark Ultra autostainer (Clone:4B5, Predilute, Ventana Medical System Inc, Roche, Tucson, Arizona) according to the BenchMark ULTRA advanced staining system operator guide. Antigen retrieval was conducted with ULTRA Cell Conditioning Solution (ULTRA CC1, pH = 8.5) at 90 to 100 °C.

The HER2 IHC score was evaluated according to the ASCO/CAP guideline. A score of 0 represents no staining or membrane staining that is incomplete and is faint/barely perceptible and in ≤10% of tumor cells; 1 + represents incomplete membrane staining that is faint/barely perceptible and in >10% of tumor cells; 2 + represents weak to moderate complete membrane staining observed in >10% of tumor cells; and 3 + represents circumferential membrane staining that is complete, intense, and in >10% of tumor cells.^12^ Also, gene amplification of HER2 was evaluated by fluorescence in situ hybridization as well.

### Statistical analysis

SPSS 29.0 (IBM, Armonk, NY, USA) and Prism 10 (GraphPad Software Inc., San Diego, CA, USA) were used for statistical analysis. Descriptive statistics were used to summarize patient characteristics, treatment administration, and antitumor activity. OS and PFS were analyzed using the Kaplan–Meier method and Cox proportional hazard models.

#### Toxicity

Toxicity data were collected and graded according to CTCAEv5.0.

## Results

### Baseline characteristics

We screened 111 patients who were diagnosed with extramammary Paget’s disease.

**Figure 1.**
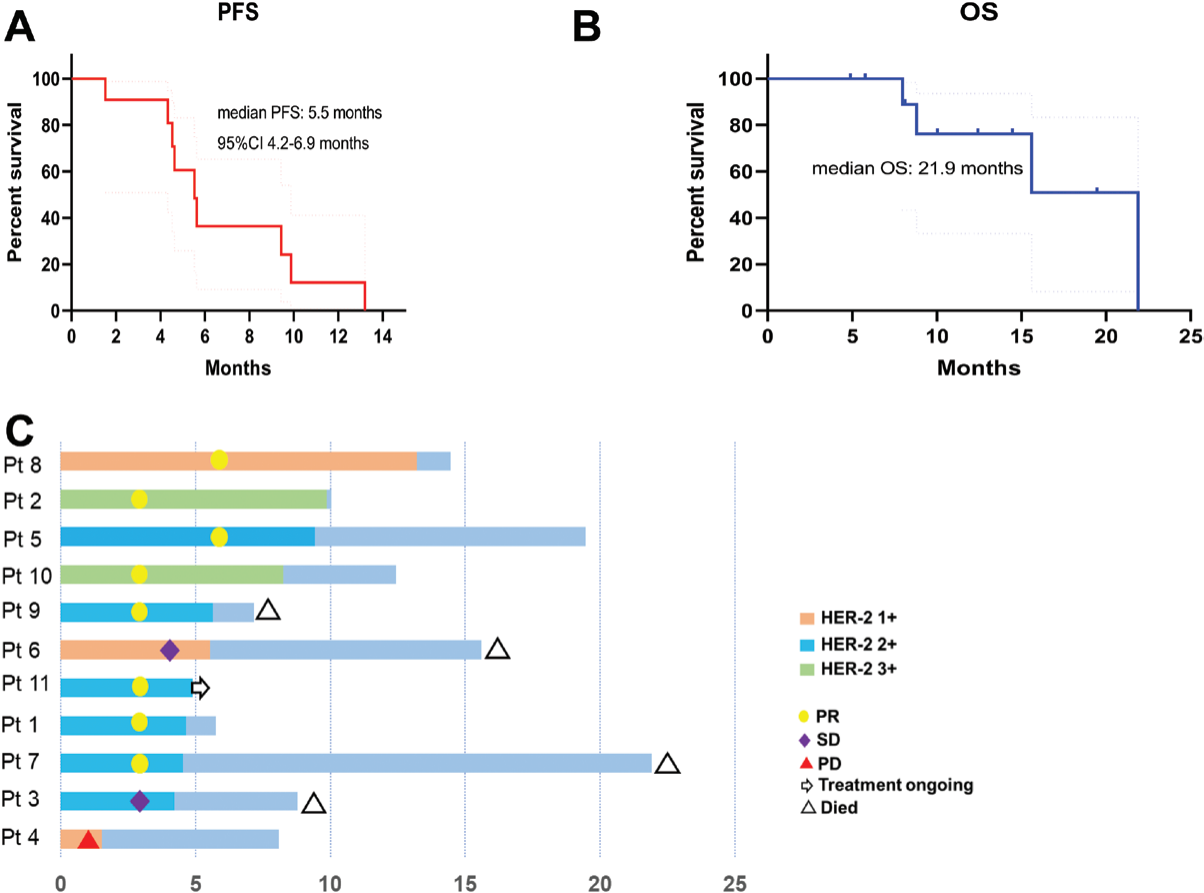
Clinical outcomes of Disitamab Vedotin treatment. A) and (B) are the curves of PFS and OS, respectively. (C) is the swimming plot of survival which denoted the HER-2 expression and treatment response.

Eleven patients were treated with DV, with efficacy and toxicity data available. Eight of eleven patients were 2 + positive for HER-2 and the other 3 patients had 1 + HER-2 staining (Figure 1). Only one patient has gene amplification (Table 1). Representative images of the HER2 IHC staining were provided in Supplementary Figure 1. The median age was 65 years (IQR 51–82) and 8 patients received DV as a second or later line of treatment (73%). All patients had distant lymph node metastases. Other metastatic sites included bone, liver, lung, adrenal gland, and spinal cord.

**Table 1.** Baseline characteristics of patients before Disitamab Vedotin treatment.

Patient number	Age	ECOG	HGB(g/L)	HER-2 (IHC)	FISH	Metastasis site	Prior treatment	Evaluation criterion
1	66	2	88	2+	Negative	lymph node, lung, liver, bone	L1: S-1 + irinotecan + bevacizumab	RECIST 1.1
2	64	0	140	3+	Negative	lymph node, liver, bone, adrenal gland	/	RECIST 1.1
3	52	2	98	2+	Negative	lymph node, bone	L1: 5-FU + cisplatin + albumin-paclitaxel + PD1	RECIST 1.1
4	70	1	134	1+	/	lymph node, bone, spinal cord	L1: Bevacizumab + irinotecan + capecitabine	RECIST 1.1
5	51	1	153	2+	Negative	lymph node	L1: Bevacizumab + irinotecan + capecitabine	Skin lesion (WHO criteria)
6	57	1	112	1+	/	lymph node, bone	L1: Bevacizumab + irinotecan + capecitabine	Skin lesion (WHO criteria)
7	73	0	142	2+	Negative	lymph node, bone	/	Skin lesion (WHO criteria)
8	75	1	140	1+	/	lymph node	L1: Bevacizumab + irinotecan	Skin lesion (WHO criteria)
9	60	1	110	2+	Negative	lymph node, liver	L1: Docetaxel L2: Liver TACE L3: Bevacizumab + irinotecan + capecitabine	RECIST 1.1
10	66	1	103	3+	Positive	lymph node, bone	L1: 5-FU + oxaliplatin L2: Docetaxel + cisplatin + PD1	RECIST 1.1
11	82	0	133	2+	Negative	lymph node, bone	/	Skin lesion (WHO criteria)

### Clinical outcome

The median follow-up was 12.4 months (95% CI: 6.4-18.5 months). Six of the eleven patients had measurable disease at baseline and were evaluated according to RECIST 1.1, and the other 5 patients were evaluated according to the skin surface change by WHO criteria. In total, 8 patients (73%) had a partial response. Two patients (18.2%) had stable disease, and only 1 patient (9.1%) had progression disease.1 patient quickly progressed after 2 cycles of DV.

**Figure 2.**
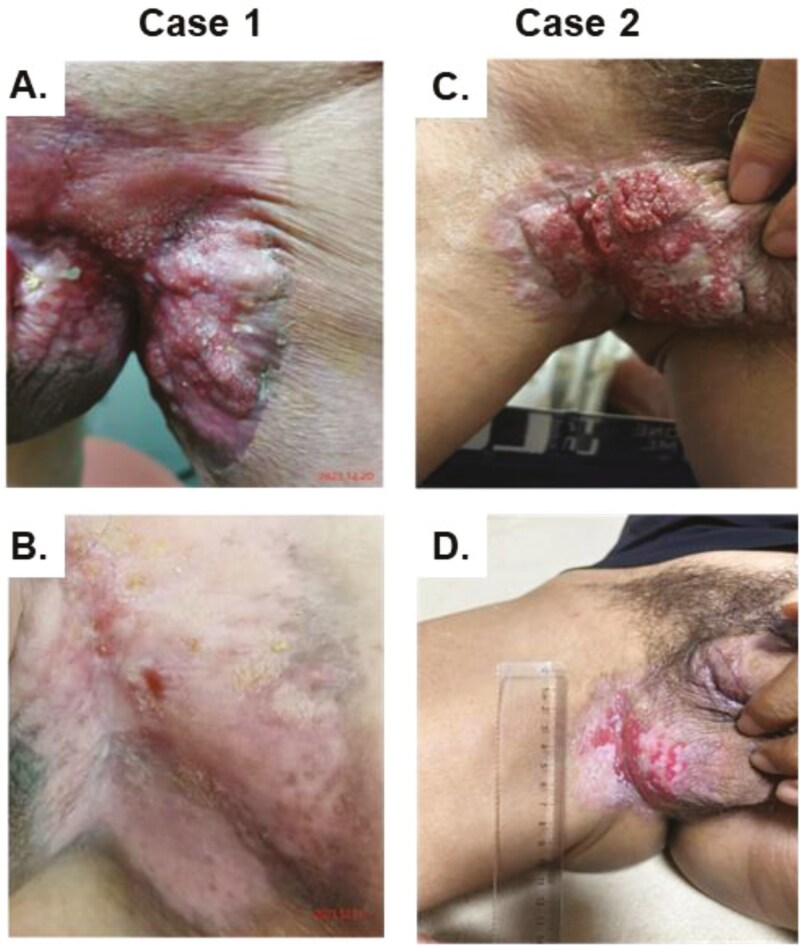
Two typical cases of response to Disitamab Vedotin treatment. Case 1 is patient 11, who has achieved a complete response in a penoscrotal skin lesion which disappeared from 7 cm, (A) and (B) are the photos before and after treatment, and this patient is still on treatment. Case 2 is patient 8, who has achieved partial response from 4.7 to 2.1 cm, (C) and (D) are the photos before and after treatment, and this patient derived 13.2 months of PFS.

In Figure 2, we show 2 cases of response to DV treatment. The responses can be easily judged through vision.

At the time of analysis, 10 of the 11 patients had exhibited progressive disease and 1 patient remains on treatment of DV. The most common progression site was in lymph nodes, followed by skin lesions, bone, lung, and the adrenal gland (Table 1). The median progression-free interval was 5.5 months (95% CI: 4.2-6.9 months; Figure 2A). The median overall survival was 21.9 months (Figure 2B).

The blood CEA level was increased in all 11 patients at baseline before DV treatment. CEA data before, on-, and after treatment were available in 9 patients: CEA decreased by more than half on treatment in 8 patients, including 6 patients with PR (Supplementary Figure 2).

Toxicity was mostly mild, and no grade 3 or higher treatment-related toxicity was observed. The most common toxicity were anemia (3), increased gamma-glutamyl transferase (2), hypoproteinemia (2), and decreased platelet count (grade 1, 2 patients; Supplementary Table 1).

## Discussion

The extreme rarity of EMPD renders formal clinical trials guiding treatment as non-feasible and high-quality retrospective data provide the best available evidence. Most previous reports of treatment for metastatic EMPD included only 1 or 2 cases, with trastuzumab and chemotherapy used commonly.^4,5,8,9,11^

Considering the toxic burden of chemotherapy for these patients, who are frequently old and frail, newer targeted therapies are needed. In contrast to previous studies showing that HER2 positivity is expressed in 30% to 60% of EMPD tumors, we found that most EMPD (95%) tumor cells express HER2, although some of them have only 1 + HER2 staining (data not shown,). This scenario has suggested the prevalence of HER2 expression and the possible use of antibody-drug conjugate targeting the HER2 pathway.

Low HER2 expression may be enough to trigger the antitumor activity of anti-HER2 antibody-drug conjugates. For example, in the treatment of breast cancer, women with ultra-low expression of HER2 have been shown to benefit from trastuzumab deruxtecan treatment.^14^ Antibody-drug conjugates have changed the treatment paradigms for several types of cancer. They generally have high target specificity and manageable toxicity.

Our group of 11 patients treated with an antibody-drug conjugate comprises one of the largest patient series with this extremely rare cancer. The response rate is relatively high.

The median progression-free interval of 5.5 months is relatively short, but these patients had received multiple lines of prior therapy. Two patients stopped DV treatments because of financial toxicity, and 1 patient remains on DV treatment without progression. PFS is expected to be longer if DV were used as first-line treatment and was continued in patients without progressive disease.

Toxicity was generally grade 1 or 2, with no grade 3 toxicity observed. This toxicity differs from reports of previous studies of DV,^12^ especially for neurotoxicity. In the current series, DV was given every 3 to 4 weeks, in comparison to every 2 weeks in clinical trials. The lower dose may account for the lower toxicity, but may also compromise the efficacy.

Another alternative treatment would be to use Trastuzumab deruxtecan, since the 1 patient quickly progressed after DV treatment, and subsequently responded well to Trastuzumab deruxtecan. However, the efficacy and toxicity of these antibody-drug conjugates should be validated in future trials.

This study is limited by its small sample size, its retrospective nature, and data missing for some patients. Selection bias occurred also because of some patients’ inability to further pay for the drug. Moreover, Inguinal and scrotal lesions are known to be difficult to assess accurately because their size changes with posture. This is also a limitation in this paper. Although the data from these male patients who have penoscrotal metastases, the finding should also be used in female patients with Paget’s disease. It has been reported that HER2 can also be expressed in female patients with Paget’s disease.^6^

We are initiating a phase 2 trial to evaluate MRG 002 (another anti-HER2 antibody-drug conjugate) for HER2-positive advanced Paget’s disease. It is anticipated that results will only become available for quite some years in the future because of the enrollment of men with an extremely rare cancer. Anti-HER2 treatment might be a choice for these patients, based on current data.

## Supplementary Material

oyaf063_suppl_Supplementary_Figures_e1

oyaf063_suppl_Supplementary_Figures_2

oyaf063_suppl_Supplementary_Tables_1

## Data Availability

The data will be available from the corresponding authors upon reasonable request.
