# Supplementary figures and images for: Antibody-drug conjugate (disitamab vedotin) therapy targeting HER2-low or higher advanced extramammary Paget’s disease
Source: Oncologist. 2025 May 27;30(5):oyaf063. doi: 10.1093/oncolo/oyaf063 (PMC12107538; doi:10.1093/oncolo/oyaf063)

## Slide 1
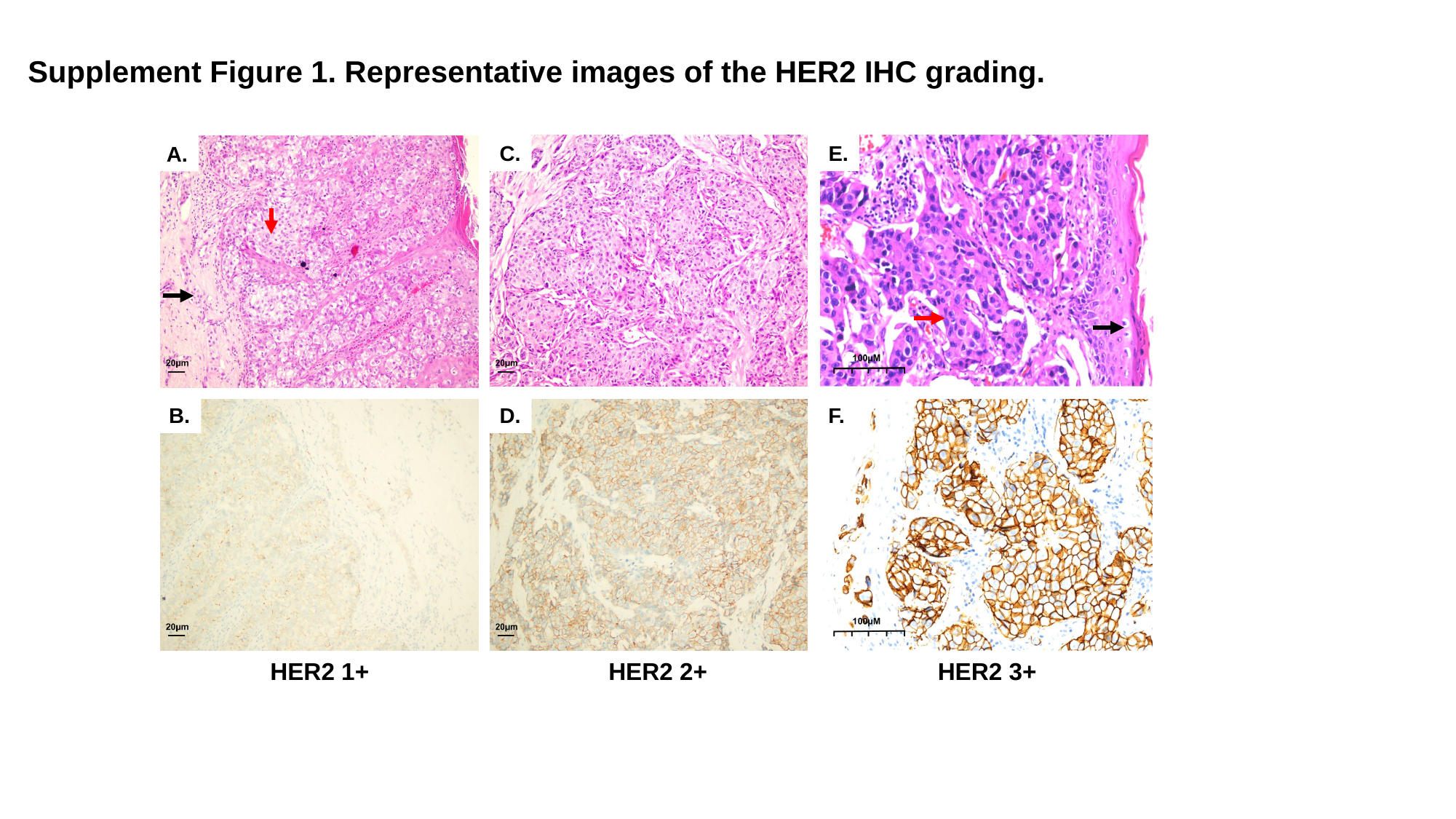

Supplement Figure 1. Representative images of the HER2 IHC grading.
C.
E.
A.
D.
F.
B.
HER2 1+
HER2 2+
HER2 3+

Supplement: oyaf063_suppl_Supplementary_Figures_e1 [file oyaf063_suppl_supplementary_figures_e1.pptx]
