# Supplementary material for: Antibody-drug conjugate (disitamab vedotin) therapy targeting HER2-low or higher advanced extramammary Paget’s disease
Source: Oncologist. 2025 May 27;30(5):oyaf063. doi: 10.1093/oncolo/oyaf063 (PMC12107538; doi:10.1093/oncolo/oyaf063)
Supplement: oyaf063_suppl_Supplementary_Figures_2 [file oyaf063_suppl_supplementary_figures_2.pptx]

## Slide 1
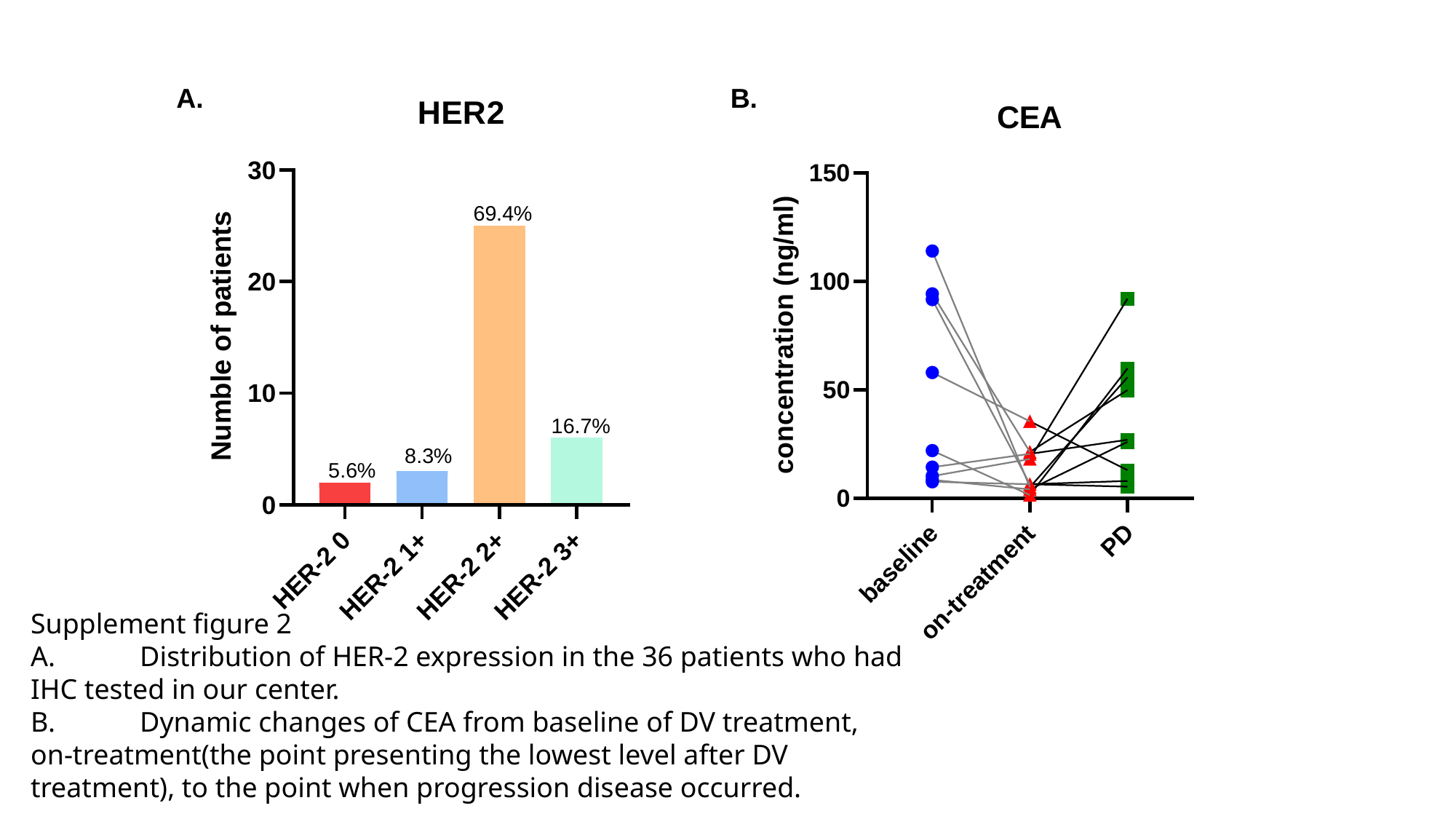

A.
B.
Supplement figure 2
A.	Distribution of HER-2 expression in the 36 patients who had IHC tested in our center.
B.	Dynamic changes of CEA from baseline of DV treatment, on-treatment(the point presenting the lowest level after DV treatment), to the point when progression disease occurred.
