# Supplementary material for: Antibody-drug conjugate (disitamab vedotin) therapy targeting HER2-low or higher advanced extramammary Paget’s disease
Source: Oncologist. 2025 May 27;30(5):oyaf063. doi: 10.1093/oncolo/oyaf063 (PMC12107538; doi:10.1093/oncolo/oyaf063)
Supplement: oyaf063_suppl_Supplementary_Tables_1 [file oyaf063_suppl_supplementary_tables_1.docx]

| **Adverse event (Grade 1-2)** | **Number of patients** |
| --- | --- |
| Hemoglobin decreased | 3 |
| Hypoproteinemia | 2 |
| Platelet count decreased | 2 |
| Gamma-glutamyl transferase increased | 2 |
| AST increased | 1 |
| Peripheral sensory neuropathy | 2 |
| Asthenia | 2 |
| Decreased appetite | 2 |
| Nausea | 2 |
| Rash | 1 |
| ALT increased | 1 |
| Leukopenia | 1 |
| Neutropenia | 1 |
| Creatinine elevation | 1 |
| Pyrexia | 1 |

Supplement table 1

Treatment-related adverse events of all patients. No one experienced serious AE and termination of treatment due to AE were not happened. All AE were well-manageable.
